# Caring for Grandchildren and Dementia Among Older Adults in China

**DOI:** 10.1001/jamanetworkopen.2025.19622

**Published:** 2025-07-09

**Authors:** Zi Zhou, Xiaoling Hong

**Affiliations:** 1 School of Public Affairs, Xiamen University, Xiamen, China

## Abstract

**Importance:**

The association between grandchild care and dementia remains unclear, with previous studies yielding mixed results and unclear mechanisms.

**Objective:**

To examine the association between grandchild care and dementia odds and to explore the mediating roles of mobile telephone ownership, broadband internet access, and reduced loneliness.

**Design, Setting, and Participants:**

This cohort study used data from the China Health and Retirement Longitudinal Study from 2013 to 2018. Data analysis was conducted from March 10, 2024, to April 20, 2025. Multistage, stratified, cluster sampling was used to recruit participants from 28 provinces across China. Data were collected through biennial, computer-assisted personal interviews. The analytic sample comprised Chinese adults aged 50 to 79 years who were dementia free at baseline.

**Exposure:**

Grandchild caregiving was classified into 4 groups: noncaregivers (0 hours per week), nonintensive caregivers (1-39 hours per week), intensive caregivers (≥40 hours per week), and those without grandchildren.

**Main Outcomes and Measures:**

Dementia was assessed using both self-reports and proxy measures, with dementia defined by scores of 0 to 6 on the modified Telephone Interview for Cognitive Status or a score of 4 or higher on the Informant Questionnaire on Cognitive Decline in the Elderly.

**Results:**

From an initial cohort of 18 605 participants, 10 058 were included (5062 men [50.3%]; 4996 women [49.7%]; mean [SD] age, 60.9 [7.2] years). Nonintensive grandchild caregiving was associated with lower odds of dementia (odds ratio [OR], 0.76; 95% CI, 0.60-0.97) than no grandchild care. The mediation analysis revealed that the association of nonintensive grandchild care with dementia odds was partially mediated by mobile telephone ownership (17.68%; 95% CI, 2.05%-37.23%), broadband internet access (17.36%; 95% CI, 5.37%-30.05%), and reduced loneliness (16.83%; 95% CI, 4.52%-30.24%), with a combined mediating proportion of 36.99% (95% CI, 25.01%-51.41%). Neither intensive grandchild caregiving nor having no grandchildren was associated with dementia odds, and no significant indirect effects were observed through the mediators examined.

**Conclusions and Relevance:**

In this cohort study of older Chinese adults, nonintensive grandchild caregiving was associated with lower odds of dementia, partly owing to increased digital technology access and reduced loneliness. Promoting digital inclusion and social engagement could be beneficial for cognitive health, particularly among older adults providing moderate levels of grandchild care.

## Key Points

**Question:**

Is grandchild caregiving associated with dementia, and are technology access and loneliness mediators of such an association?

**Findings:**

In this cohort study of 10 058 older adults, nonintensive grandchild caregiving was significantly associated with reduced odds of dementia, and this association was partially mediated by mobile telephone ownership (17.68% mediation), broadband internet access (17.36% mediation), and low loneliness (16.83% mediation).

**Meaning:**

These findings reveal the pathways by which grandchild caregiving is associated with dementia odds through digital technology access and low loneliness and underscore the potential of promoting digital literacy and fostering social engagement to mitigate the odds of dementia among older adults.

## Teaser

This cohort study examines the association between grandchild care and dementia odds and explores the mediating roles of mobile telephone ownership, broadband internet access, and reduced loneliness.

## Introduction

Dementia is a pressing public health concern, with a substantial burden in China, which accounts for nearly 25% of worldwide cases and great socioeconomic costs.^1,2,3^ This fact highlights the urgent need for effective prevention strategies.^1^ Although associations between dementia risk and modifiable factors like social engagement and loneliness are known,^4,5,6^ the role of grandchild caregiving—a common practice in China driven by cultural norms—remains less clear.^7^ Research on the association between grandchild caregiving and cognitive function yields mixed findings, with some studies suggesting a protective association^8,9,10,11^ and others reporting null or even negative associations.^12^ The complexity of this association underscores the need for further investigation into its underlying mechanisms.

The cognitive reserve hypothesis suggests that caregiving activities, such as planning, teaching, and interacting with grandchildren, may directly promote executive function, memory, and problem-solving skills, thereby building cognitive resilience and delaying dementia onset.^13,14,15^ However, caregiving intensity may be differentially associated with dementia risk owing to varying levels of stress, physical demands, and social engagement.^16^ These distinctions highlight the importance of examining caregiving intensity when assessing the association between grandchild caregiving and dementia risk.

In addition to direct cognitive associations, caregiving may influence dementia risk through indirect pathways, such as technology access and reductions in loneliness, which may hold particular importance in today’s increasingly digital and interconnected world.^17^ Grandchild caregiving can foster older adults’ engagement with digital devices, such as mobile telephones or broadband internet, to maintain family communication.^18,19^ This engagement with technology, which requires learning new skills while promoting social connection and cognitive stimulation,^20,21^ has been linked to better cognitive outcomes in older adults.^20,22^

Another critical pathway is loneliness.^23^ Grandchild caregiving may help mitigate loneliness by fostering meaningful social roles and increasing intergenerational interactions, thereby promoting emotional well-being.^24,25^ Drawing on social integration theory, caregiving may provide a sense of purpose and connection, thus buffering against the detrimental associations of loneliness with cognitive health.^26^ Despite plausible pathways, rigorous research directly examining the mediating roles of technology access and loneliness in the caregiving-dementia relationship is lacking.

This study used 3 waves (2013, 2015, and 2018) of the nationally representative China Health and Retirement Longitudinal Study (CHARLS) to examine the direct and indirect effects of grandchild caregiving on dementia odds, focusing on mediation by technology access and loneliness. To address time-varying confounding in this longitudinal analysis, we used marginal structural models (MSMs).^27^ Complementarily, we use inverse odds ratio weighting (IORW) for mediation analysis to estimate the natural direct effect of caregiving and the indirect effects via technology access and loneliness.^28^ These findings aim to clarify the cognitive impacts of caregiving and inform targeted interventions for dementia prevention. The Figure shows a directed acyclic graph of the hypothesized relationships.

**Figure.**
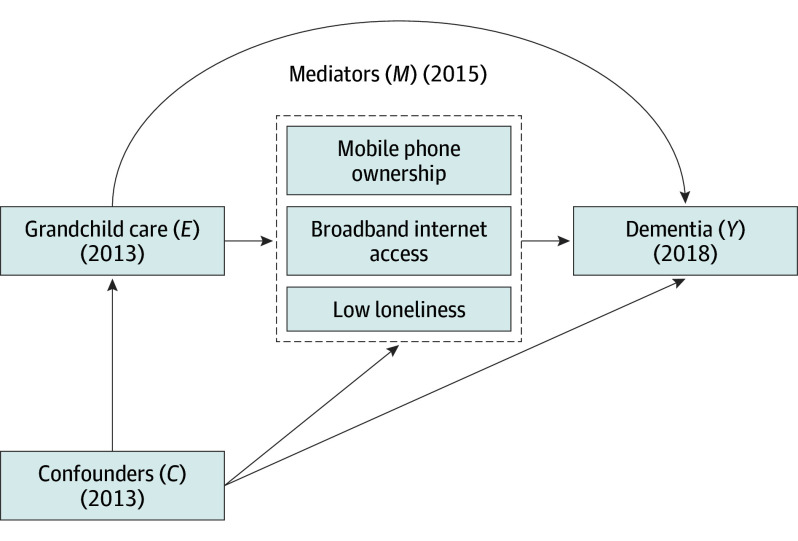
Directed Acyclic Graph Illustrating the Proposed Mediating Pathways Linking Grandchild Care to Dementia

## Methods

### Data and Sample

This study used data from the CHARLS, a nationally representative survey of Chinese adults aged 45 years and older. CHARLS used a multistage, stratified, cluster sampling approach to select participants from 28 provinces across China. The CHARLS sample was randomly selected through a probability proportional to the size sampling method. The participants were interviewed every 2 years via computer-assisted personal interviewing techniques. The study was approved by the institutional review board of Peking University, and all participants provided written informed consent before participating. Detailed information on the CHARLS sampling methods and data quality procedures has been published elsewhere.^29^ This study follows the Strengthening the Reporting of Observational Studies in Epidemiology (STROBE) reporting guidelines.

For our analysis, we used data from the 2013 baseline (18 605 participants) and subsequent waves in 2015 and 2018. Our analytic sample excluded individuals younger than 50 years (3229 participants) because caregiving responsibilities for grandchildren are extremely rare in this age group. In addition, we excluded individuals aged 80 years and older (670 participants) because of the greater likelihood of severe health limitations impacting their caregiving capacity. We further excluded those who reported dementia at baseline or at the first follow-up (1401 participants) to minimize reverse causation bias; those who died or were lost to follow-up between 2013 and 2018 (2462 participants); and those with missing data on key variables, such as outcomes, exposures, and mediators (785 participants). The sample selection process is illustrated in the eFigure in Supplement 1.

### Measure

#### Outcome

Dementia was assessed in the 2018 wave of the CHARLS by using both self-reports and proxy measures to account for potential participant limitations.^30^ For participants able to self-report the Telephone Interview for Cognitive Status and its modifications (TICS-m),^31^ this validated instrument encompasses assessments of episodic memory (10-word immediate and delayed recall; score range, 0-20), working memory (serial 7’s subtraction; score range, 0-5), and attention (backward counting; score range, 0-2).^32^ These subscores were summed to generate a composite cognitive score (range, 0-27), with lower scores indicating poorer cognitive performance. In accordance with the Langa-Weir criteria, participants scoring 0 to 6 were classified as having dementia.^33,34^

For participants unable to complete the TICS-m because of health constraints, dementia status was assessed via the Informant Questionnaire on Cognitive Decline in the Elderly.^35^ This 26-item questionnaire, administered to a knowledgeable informant (eg, family member), captures longitudinal changes in the participant’s cognitive function and activities of daily living over the preceding decade. A mean score across all items was calculated, with scores of 4 or higher indicative of dementia, adhering to established thresholds.^36^

#### Exposure

Grandchild caregiving status and intensity were assessed using data from the CHARLS 2013. Participants indicated whether they had living grandchildren, whether they provided care in the past year, and the mean weekly hours involved. On the basis of these responses, participants were classified into 4 mutually exclusive exposure groups: (1) noncaregivers (had grandchildren, 0 hours per week care), (2) nonintensive caregivers (1-39 hours per week), (3) intensive caregivers (≥40 hours per week), and (4) having no grandchildren. The hourly cutoffs for nonintensive and intensive caregiving align with previous research,^16,37^ with 40 hours or more per week representing a substantial commitment comparable to full-time employment.

### Mediators

The mediators, including mobile telephone ownership, broadband internet access, and low loneliness, were measured during wave 4 (2015). Mobile telephone ownership was assessed via a single self-reported item, with participants who owned a mobile telephone, including both basic mobile telephones and smartphones, classified as owners (1) and those who did not as nonowners (0).^38^ Broadband internet access was evaluated via a similar self-reported measure, with participants who had a broadband internet connection classified as having access (1) and those who did not have access (0). Although these measures do not capture the full complexity of technology access, they provide a practical method for assessing basic access.^21^

Loneliness was evaluated via a single-item indicator of self-reported loneliness frequency. Participants who responded rarely or a little of the time were coded as having low loneliness (1), whereas those indicating a moderate amount or most of the time were classified as experiencing loneliness (0). Although acknowledging the inherent limitations of single-item assessments, this approach is widely used in large-scale studies and has established psychometric properties.^39^

### Covariates

The study’s covariates encompass 3 main domains: sociodemographic factors, behavioral characteristics, and health indicators. Specifically, sociodemographic factors include age (50-59, 60-69, and 70-79 years), gender, ethnic background (Han or minoritized group), residency (rural or urban), marital status, educational level (nonliterate, elementary school, and middle school or above), current occupational status, log-transformed income, and living arrangements (categorized as living with spouse only, skipped-generation residence, living alone, or other). The behavioral characteristics included current smoking status (current, former, or never), current drinking status (current, former, or never), and sleep duration per night (<6, 6-9, or 9 hours). Health indicators were assessed by medical insurance, the presence of any chronic diseases, and activities of daily living disabilities.

### Statistical Analysis

Data analysis was conducted from March 10, 2024, to April 20, 2025. We began by summarizing participant characteristics via descriptive statistics. Continuous and categorical variables were compared using analysis of variance and χ^2^ tests, respectively. We used a 2-stage MSMs approach to evaluate the associations between grandchild caregiving and dementia odds and the potential mediating roles.^27^ We used IORW, which is well suited for dissecting the pathways within a multiple mediator model^28^ (for details, see the eMethods in Supplement 1).

To ensure the robustness of our findings, we implemented several key sensitivity analyses. First, we calculated E-values to quantify the potential impact of unmeasured confounding variables. E-values provide an estimate of the minimum strength of association that an unobserved confounder would need to have with both the exposure and the outcome to explain away the observed association.^40^ Second, because MSMs can be sensitive to extreme weights, we implemented alternative truncation ranges for the stabilized weights, specifically the 5th to 95th and 10th to 99th percentiles.^41^ Third, sensitivity analyses assessed robustness using alternative definitions for intensive grandchild caregiving: 30 hours or more per week, and the upper tertile and upper quartile of caregiving hours. Fourth, to account for discrepancies between broadband access and actual usage, we used an alternative self-reported measure of internet use. Finally, to assess potential bias from the dementia assessment method, we restricted analyses to participants with self-reported TICS-m dementia status and repeated the primary analysis.

Missing data in our longitudinal analysis were addressed via a combined approach. For instances of missing exposure, mediators and outcome data, we used the last observation carried forward method. Missing confounder data were handled via multiple imputation techniques.^42^ Statistical analyses were performed using Stata statistical software version 16.0 (StataCorp). Two-sided *P* < .05 indicated statistical significance.

## Results

Overall, of the 10 058 included participants, 5062 (50.3%) were men, 4996 (49.7%) were women, and the mean (SD) age was 60.9 (7.2) years. As shown in Table 1, participants engaging in nonintensive grandchild caregiving exhibited distinct sociodemographic characteristics compared with those in the other groups. Individuals in the nonintensive caregiving group were more likely to be married and currently working. They also were more likely to report lower levels of loneliness. Notably, the prevalence of dementia was significantly lower among those providing nonintensive grandchild care (130 participants [7.2%]) than among all the nongrandchild care (439 participants [10.7%]), intensive grandchild care (173 participants [7.8%]), and having no grandchild (147 participants [7.6%]) groups. In the weighted population, the analysis revealed that covariates and confounders were well balanced across different levels of grandchild caregiving (eTable 1 in Supplement 1).

**Table 1.** Characteristics of the Participants According to Grandchild Care in the Analytic Sample

Characteristics	Participants, No. (%)					*P* value
	Total (N = 10 058)	Noncaregivers (n = 4106)	Nonintensive caregivers (n = 1813)	Intensive caregivers (n = 2216)	Having no grandchild (n = 1923)	
Age, mean (SD), y						
50-59	4684 (46.6)	1528 (37.2)	888 (49.0)	1117 (50.4)	1151 (59.9)	<.001
60-69	3969 (39.5)	1813 (44.2)	761 (42.0)	945 (42.6)	450 (23.4)	
70-79	1405 (14.0)	765 (18.6)	164 (9.1)	154 (7.0)	322 (16.7)	
Gender						
Men	5062 (50.3)	2181 (53.1)	909 (50.1)	940 (42.4)	1032 (53.7)	<.001
Women	4996 (49.7)	1925 (46.9)	904 (49.9)	1276 (57.6)	891 (46.3)	
Ethnicity						
Han	9333 (92.8)	3833 (93.4)	1662 (91.7)	2075 (93.6)	1763 (91.7)	.01
Minoritized group	725 (7.2)	273 (6.6)	151 (8.3)	141 (6.4)	160 (8.3)	
Rural residence	7719 (76.7)	3371 (82.1)	1396 (77.0)	1772 (80.0)	1180 (61.4)	<.001
Married	8987 (89.4)	3642 (88.7)	1662 (91.7)	2025 (91.4)	1658 (86.2)	<.001
Education						
Nonliterate	2263 (22.5)	1020 (24.8)	414 (22.8)	541 (24.4)	288 (15.0)	<.001
Elementary school	4258 (42.3)	1862 (45.4)	765 (42.2)	954 (43.1)	677 (35.2)	
Middle school or above	3537 (35.2)	1224 (29.8)	634 (35.0)	721 (32.5)	958 (49.8)	
Current working	7004 (69.6)	2903 (70.7)	1306 (72.0)	1525 (68.8)	1270 (66.0)	<.001
Log-transformed annual income, mean (SD), yuan^a^	8.0 (2.4)	8.0 (2.3)	7.9 (2.4)	8.0 (2.3)	8.2 (2.3)	.08
Living arrangements						
Living with spouse only	3026 (30.1)	1679 (40.9)	375 (20.7)	402 (18.1)	570 (29.6)	<.001
Skipped-generation residence	935 (9.3)	162 (4.0)	300 (16.6)	429 (19.4)	44 (2.3)	
Living alone	541 (5.4)	303 (7.4)	42 (2.3)	46 (2.1)	150 (7.8)	
Other	5556 (55.2)	1962 (47.8)	1096 (60.5)	1339 (60.4)	1159 (60.3)	
Smoking status						
Nonsmoker	5553 (55.2)	2137 (52.1)	1005 (55.4)	1365 (61.6)	1046 (54.4)	<.001
Former smoker	714 (7.1)	335 (8.2)	117 (6.5)	127 (5.7)	135 (7.0)	
Current smoker	3791 (37.7)	1634 (39.8)	691 (38.1)	724 (32.7)	742 (38.6)	
Drinking status						
Nondrinker	5354 (53.2)	2140 (52.1)	968 (53.4)	1273 (57.5)	973 (50.6)	<.001
Former drinker	1072 (10.7)	464 (11.3)	181 (10.0)	237 (10.7)	190 (9.9)	
Current drinker	3632 (36.1)	1502 (36.6)	664 (36.6)	706 (31.9)	760 (39.5)	
Sleep duration per night, h						
<6	3414 (33.9)	1417 (34.5)	582 (32.1)	792 (35.7)	623 (32.4)	.001
6-9	6044 (60.1)	2405 (58.6)	1121 (61.8)	1314 (59.3)	1204 (62.6)	
≥9	600 (6.0)	284 (6.9)	110 (6.1)	110 (5.0)	96 (5.0)	
Medical insurance	9752 (97.0)	3986 (97.1)	1771 (97.7)	2156 (97.3)	1839 (95.6)	.001
Chronic conditions	7319 (72.8)	3036 (73.9)	1338 (73.8)	1639 (74.0)	1306 (67.9)	.001
Activities of daily living disabled	1221 (12.1)	590 (14.4)	183 (10.1)	254 (11.5)	194 (10.1)	<.001
Mobile telephone ownership	9072 (90.2)	3600 (87.7)	1666 (91.9)	2062 (93.1)	1744 (90.7)	<.001
Broadband internet access	2541 (25.3)	760 (18.5)	500 (27.6)	596 (26.9)	685 (35.6)	<.001
Low loneliness	8470 (84.2)	3391 (82.6)	1572 (86.7)	1884 (85.0)	1623 (84.4)	<.001
Dementia	889 (8.8)	439 (10.7)	130 (7.2)	173 (7.8)	147 (7.6)	<.001

^a^ As of June 6, 2025, 1.00 yuan = $0.14 US.

Grandparents providing nonintensive care were more likely than noncaregivers to own a mobile telephone (odds ratio [OR], 1.33; 95% CI, 1.06-1.67), have access to broadband internet (OR, 1.34; 95% CI, 1.10-1.62), and experience reduced loneliness (OR, 1.26; 95% CI, 1.04-1.52) (eTable 2 in Supplement 1). Table 2 presents the associations between grandchild care and dementia odds among older Chinese adults, along with the mediating effects of mobile telephone ownership, broadband internet access, and low loneliness, using MSMs. Nonintensive grandchild care was consistently associated with a reduced odds of dementia across all the models. In model 1, the OR was 0.69 (95% CI, 0.54-0.88), and in model 5, the OR was 0.76 (95% CI, 0.60-0.97). Conversely, neither intensive grandchild care nor having no grandchild was significantly associated with dementia odds in any model. Mobile telephone ownership, broadband internet access, and reduced loneliness were consistently associated with lower dementia odds across all the models.

**Table 2.** Association of Grandchild Care With Dementia Risk and Mediating Associations Among 10 058 Chinese Older Adults According to Marginal Structural Models

Variable	OR (95% CI)^a^				
	Model 1^b^	Model 2^c^	Model 3^d^	Model 4^e^	Model 5^f^
Nonintensive grandchild care	0.69 (0.54-0.88)	0.74 (0.58-0.94)	0.73 (0.57-0.92)	0.71 (0.56-0.90)	0.76 (0.60-0.97)
Intensive grandchild care	0.82 (0.66-1.02)	0.84 (0.68-1.04)	0.85 (0.69-1.06)	0.84 (0.68-1.04)	0.86 (0.69-1.07)
Having no grandchild	1.11 (0.87-1.40)	1.16 (0.91-1.48)	1.17 (0.92-1.49)	1.12 (0.89-1.43)	1.21 (0.95-1.55)
Mobile telephone ownership	NA	0.66 (0.52-0.84)	NA	NA	0.70 (0.55-0.90)
Broadband internet access	NA	NA	0.45 (0.35-0.57)	NA	0.43 (0.33-0.57)
Low loneliness	NA	NA	NA	0.58 (0.48-0.71)	0.64 (0.52-0.78)
Age	1.92 (1.71-2.15)	1.83 (1.63-2.07)	1.83 (1.62-2.06)	1.92 (1.71-2.15)	1.72 (1.52-1.94)
Men	0.49 (0.41-0.58)	0.49 (0.40-0.58)	0.49 (0.41-0.59)	0.50 (0.42-0.60)	0.50 (0.41-0.60)
Ethnic Han	0.61 (0.47-0.80)	0.61 (0.47-0.80)	0.65 (0.50-0.86)	0.62 (0.47-0.81)	0.67 (0.51-0.88)
Constant	0.12 (0.09-0.17)	0.18 (0.12-0.26)	0.13 (0.10-0.18)	0.18 (0.13-0.25)	0.27 (0.18-0.40)

Abbreviations: NA, not applicable; OR odds ratio.

^a^ Stabilized weights accounted for residency, marital status, education, current working status, income, living arrangements, smoking status, drinking status, sleep duration, medical insurance, chronic conditions, and activities of daily living disabilities.

^b^ Model 1 adjusted for age, men, and Han ethnicity.

^c^ Model 2 further adjusted for mobile telephone ownership based on model 1.

^d^ Model 3 further adjusted for broadband internet access based on model 1.

^e^ Model 4 further adjusted for low loneliness based on model 1.

^f^ Model 5 further adjusted for mobile telephone ownership, broadband internet access, and low loneliness based on model 1.

Mediation analyses using IORW provided additional insights into the potential pathways linking caregiving with dementia odds (Table 3). The direct protective association of nonintensive caregiving with dementia odds (natural direct effect OR, 0.74; 95% CI, 0.66-0.81) remained significant, even after accounting for mediators. The proportions of the total effect mediated were 17.68% (95% CI, 2.05%-37.23%) for mobile telephone ownership, 17.36% (95% CI, 5.37%-30.05%) for broadband internet access, and 16.83% (95% CI, 4.52%-30.24%) for reduced loneliness, with a combined mediating proportion of 36.99% (95% CI, 25.01%-51.41%). However, for intensive caregiving and having no grandchild, the mediation proportions were not statistically significant.

**Table 3.** Mediating Roles of Mobile Telephone Ownership, Broadband Internet Access, and Low Loneliness in the Association Between Grandchild Care and Dementia Risk According to Inverse OR Weighted Model

Exposure and mediators	OR (95% CI)^a^			Mediation proportion, % (95% CI)^b^
	Total effect	Natural direct effects	Natural indirect effects	
Nonintensive grandchild care				
Mobile telephone ownership (M1)	0.69 (0.64 to 0.74)	0.74 (0.66 to 0.81)	0.94 (0.87 to 0.99)	17.68 (2.05 to 37.23)
Broadband internet access (M2)	0.69 (0.64 to 0.74)	0.74 (0.67 to 0.80)	0.94 (0.90 to 0.98)	17.36 (5.37 to 30.05)
Low loneliness (M3)	0.69 (0.64 to 0.74)	0.73 (0.67 to 0.80)	0.94 (0.90 to 0.98)	16.83 (4.52 to 30.24)
M1 + M2 + M3	0.69 (0.64 to 0.74)	0.79 (0.73 to 0.85)	0.87 (0.83 to 0.91)	36.99 (25.01 to 51.41)
Intensive grandchild care				
Mobile telephone ownership (M1)	0.82 (0.66 to 1.02)	0.84 (0.65 to 1.09)	0.97 (0.93 to 1.03)	14.36 (−14.84 to 41.69)
Broadband internet access (M2)	0.82 (0.66 to 1.02)	0.82 (0.65 to 1.03)	1.00 (0.96 to 1.04)	−0.25 (−20.69 to 19.15)
Low loneliness (M3)	0.82 (0.66 to 1.02)	0.82 (0.65 to 1.03)	1.01 (0.97 to 1.05)	−3.59 (−25.04 to 17.36)
M1 + M2 + M3	0.82 (0.66 to 1.02)	0.82 (0.66 to 1.02)	1.00 (0.95 to 1.04)	0.66 (−25.11 to 23.27)
Having no grandchild				
Mobile telephone ownership (M1)	1.11 (0.87 to 1.40)	1.10 (0.83 to 1.46)	1.01 (0.95 to 1.06)	6.32 (−67.23 to 111.37)
Broadband internet access (M2)	1.11 (0.87 to 1.40)	1.14 (0.88 to 1.49)	0.97 (0.93 to 1.01)	−29.66 (−144.22 to 7.88)
Low loneliness (M3)	1.11 (0.87 to 1.40)	1.12 (0.86 to 1.47)	0.98 (0.94 to 1.02)	−14.81 (−103.11 to 36.84)
M1 + M2 + M3	1.11 (0.87 to 1.40)	1.08 (0.86 to 1.36)	1.03 (0.98 to 1.07)	24.89 (−49.35 to 105.20)

Abbreviations: M, mediator; OR, odds ratio.

^a^ Stabilized weights accounted for residency, marital status, education, current working status, income, living arrangements, smoking status, drinking status, sleep duration, medical insurance, chronic conditions, and activities of daily living disabilities.

^b^ Calculated as [natural indirect effects / (natural direct effects + natural indirect effects)] × 100%.

Sensitivity analyses confirmed the robustness of these findings. First, associations between grandchild caregiving and dementia odds were stable across different weight truncation thresholds (eTables 3 and 4 in Supplement 1). Second, the E-values for nonintensive caregiving (range, 1.96-2.26) indicated moderate resilience to potential unmeasured confounding factors (eTable 5 in Supplement 1). Third, results were also consistent when alternative definitions of intensive care were used, including 30 hours per week, upper tertile, and upper quantile (eTables 6, 7, and 8 in Supplement 1). Finally, consistent results were observed in mediation analyses using self-reported internet use instead of broadband access (eTable 9 in Supplement 1) and using TICS-m for dementia assessment (eTable 10 in Supplement 1).

## Discussion

Using causal inference methods (MSMs and IORW), this cohort study found that nonintensive caregiving of grandchildren was associated with lower odds of dementia and that this protective association was partially mediated by technology access and reduced loneliness. Importantly, no significant mediation pathway was found for intensive caregiving or absence of grandchildren, suggesting that extremes in caregiving may negate potential cognitive benefits from social and digital engagement.

Our findings are consistent with those of prior studies indicating a significant association between nonintensive caregiving and reduced odds of dementia.^15^ Furthermore, this study expands on prior research by using causal inference analysis within the counterfactual framework.^27^ The protective association of nonintensive caregiving with dementia odds supports the use-it-or-lose-it hypothesis, which posits that engaging in mentally and socially stimulating activities—such as interacting with grandchildren—can preserve cognitive reserves and delay cognitive decline.^43^ These benefits, however, appear to have a threshold, as intensive caregiving, often accompanied by heightened stress and caregiving burden, was not associated with reduced dementia odds.^44^ This null finding might be influenced by socioeconomic status. Intensive caregiving driven by necessity, which is more common among individuals with lower socioeconomic status, may entail stressors (eg, financial strain) that counteract cognitive gains. This aligns with the stress process model, where chronic caregiving demands are associated with higher stress levels, erode cognitive reserves, and contribute to physiological pathways that are linked to greater odds of dementia.^44,45^

We extended previous findings by investigating the mediating role of digital technology access and low loneliness in the association between grandchild caregiving and dementia odds using IORW. One potential pathway from grandchild caregiving to dementia odds in older adults is through access to technology. We found that participants providing nonintensive care had higher odds of mobile telephone access, aligning with previous findings.^18^ This may be because nonintensive caregiving allows grandparents more time and flexibility to engage in digital communication with grandchildren and other family members.^18^ Mobile telephone ownership, as shown in prior studies,^46,47^ facilitates social interaction and cognitive stimulation, contributing to reduced dementia odds. Conversely, the absence of mediation in the intensive caregiving group suggests that heavy caregiving responsibilities may restrict time or capacity for cognitively beneficial technology use.^16^

Similarly, our study identified broadband internet access as a significant mediator. We found that grandchild caregiving was associated with broadband internet access, potentially related to needs for family communication or supporting grandchildren’s educational and social needs.^18^ In turn, access to the broadband internet provides older adults with opportunities for cognitive engagement through various online activities, such as information seeking, online games, and social networking,^20^ which may contribute to cognitive reserve.^22^ The stronger mediation effect observed for nonintensive caregivers might reflect their greater flexibility to engage with the internet for cognitive enrichment compared with potentially time-constrained intensive caregivers.

Furthermore, our findings underscore the mediating role of reduced loneliness in the association between nonintensive grandchild caregiving and dementia odds. We found that grandchild caregiving was associated with reduced loneliness. A plausible explanation is that nonintensive caregiving may foster a sense of purpose and social connection by providing opportunities for meaningful interactions with family and grandchildren, thereby buffering against loneliness, consistent with evidence on moderate caregiving enhancing well-being.^24^ Furthermore, a growing body of evidence has demonstrated that reduced loneliness is a protective factor against dementia.^48,49^ The lack of a significant mediating effect for intensive caregiving could be explained by the possibility that excessive caregiving burden might contribute to social isolation, thus counteracting the potential benefits of social interaction inherent in caregiving.

In our study, the absence of grandchildren itself appears to represent a distinct condition where the investigated mediating factors do not confer the associations between mediators and dementia risk observed in nonintensive caregivers. This could imply that the specific nature of the grandparent-grandchild relationship provides unique cognitive stimulation not easily replaced by general social or digital activities.^50^

### Limitations

This study has several limitations. First, our findings rely on MSMs assumptions, including exchangeability, positivity, and correct model specification.^41^ Although we adjusted for a range of confounders, and E-value sensitivity analyses suggested only moderate unmeasured confounding could explain the observed associations,^40^ residual confounding cannot be completely excluded, especially from factors such as cognitive engagement or social network. Second, the validity of both the positivity and correct model specification assumptions relies on the stabilized weights with means near 1.^51^ Our estimated stabilized weight means (0.99-1.00) suggest that these assumptions were likely met. Third, truncating extreme weights necessitates balancing the reduction in weight variability against the potential for residual confounding.^41^ We explored this trade-off through sensitivity analyses using various weight truncation thresholds. Fourth, our measure of grandchild caregiving relied on self-reports and did not capture the full spectrum of caregiving activities or their emotional valence. Future studies incorporating objective measures and qualitative data on caregiving experiences are needed. Fifth, although our study used validated instruments, the TICS-m and Informant Questionnaire on Cognitive Decline in the Elderly, for assessing dementia, it is important to acknowledge that these measures rely on self-reports and proxy observations. Future research could benefit from incorporating clinically based diagnostic assessments. Sixth, reverse causality could not be ruled out. However, importantly, this study excluded participants who received a diagnosis of dementia in 2015, which mitigated the issue of reverse causality. Seventh, other potential mediating factors, such as emotional well-being and stress management, warrant further investigation in future research.

## Conclusions

This cohort study provides novel insights into the complex interplay between grandchild caregiving, digital technology access, loneliness, and dementia odds among older Chinese adults. By leveraging MSMs and IORW, we found that nonintensive grandchild caregiving was associated with reduced odds of dementia, and this protective association was partially mediated by increased mobile telephone ownership, broadband internet access, and reduced loneliness. These mediating pathways underscore the importance of digital inclusion and social engagement in promoting cognitive health among older adults engaged in moderate levels of grandchild caregiving. Conversely, neither intensive grandchild caregiving nor having no grandchild showed a significant association with dementia odds. These findings underscore the importance of considering caregiving intensity and family structure when designing interventions, such as supporting moderate caregiving engagement, enhancing digital literacy and access, and strengthening social connectedness, to reduce dementia odds.

## Notes

Supplement 1. **eMethods**. Marginal structural models (MSMs) and inverse odds ratio weighting (IORW) analysis **eTable 1.** Summary of Time-varying Confounders according to Grandchild Care in the Weighted Population **eTable 2.** Association of Grandchild Care with Mobile Phone Ownership, Broadband Internet Access, and Low Loneliness in Older Chinese Adults: MSMs (N = 10,058) **eTable 3.** Mediation Analyses of the Association between Grandchild Care and Dementia Risk via MSMs with 5th/95th Truncated Weights (N = 10,058) **eTable 4.** Mediation Analyses of the Association between Grandchild Care and Dementia Risk via MSMs with 10th/90th Truncated Weights (N = 10,058) **eTable 5.** E values of the Association between Grandchild Care and Dementia Risk in Older Chinese Adults (N = 10,058) **eTable 6.** Mediating Roles of Mobile Phone Ownership, Broadband Internet Access, and Low Loneliness on Dementia Risk via Intensive Grandchild Care Defined as 30 or More: IORW (N = 10,058) **eTable 7.** Mediating Roles of Mobile Phone Ownership, Broadband Internet Access, and Low Loneliness on Dementia Risk via Intensive Grandchild Care Defined by Upper Tertile Cutoff: IORW (N = 10,058) **eTable 8.** Mediating Roles of Mobile Phone Ownership, Broadband Internet Access, and Low Loneliness on Dementia Risk via Intensive Grandchild Care Defined by Upper Quantile Cutoff: IORW (N = 10,058) **eTable 9.** Mediating Roles of Mobile Phone Ownership, Internet Usage, and Low Loneliness on the Association between Grandchild Care and Dementia Risk: IORW (N = 10,058) **eTable 10.** Mediating Roles of Mobile Phone Ownership, Broadband Internet Access, and Low Loneliness on Dementia Risk among Participants with Dementia Assessment via TICSm: IORW (N = 9,385) **eFigure.** Sample selection flowchart
eReference

Supplement 2.
Data Sharing Statement
