# Supplementary material for: Caring for Grandchildren and Dementia Among Older Adults in China
Source: JAMA Netw Open. 2025 Jul 9;8(7):e2519622. doi: 10.1001/jamanetworkopen.2025.19622 (PMC12242682; doi:10.1001/jamanetworkopen.2025.19622)
Supplement: Supplement 1. — eMethods. Marginal structural models (MSMs) and inverse odds ratio weighting (IORW) analysis eTable 1. Summary of Time-varying Confounders according to Grandchild Care in the Weighted Population eTable 2. Association of Grandchild Care with Mobile Phone Ownership, Broadband Internet Access, and Low Loneliness in Older Chinese Adults: MSMs (N = 10,058) eTable 3. Mediation Analyses of the Association between Grandchild Care and Dementia Risk via MSMs with 5th/95th Truncated Weights (N = 10,058) eTable 4. Mediation Analyses of the Association between Grandchild Care and Dementia Risk via MSMs with 10th/90th Truncated Weights (N = 10,058) eTable 5. E values of the Association between Grandchild Care and Dementia Risk in Older Chinese Adults (N = 10,058) eTable 6. Mediating Roles of Mobile Phone Ownership, Broadband Internet Access, and Low Loneliness on Dementia Risk via Intensive Grandchild Care Defined as 30 or More: IORW (N = 10,058) eTable 7. Mediating Roles of Mobile Phone Ownership, Broadband Internet Access, and Low Loneliness on Dementia Risk via Intensive Grandchild Care Defined by Upper Tertile Cutoff: IORW (N = 10,058) eTable 8. Mediating Roles of Mobile Phone Ownership, Broadband Internet Access, and Low Loneliness on Dementia Risk via Intensive Grandchild Care Defined by Upper Quantile Cutoff: IORW (N = 10,058) eTable 9. Mediating Roles of Mobile Phone Ownership, Internet Usage, and Low Loneliness on the Association between Grandchild Care and Dementia Risk: IORW (N = 10,058) eTable 10. Mediating Roles of Mobile Phone Ownership, Broadband Internet Access, and Low Loneliness on Dementia Risk among Participants with Dementia Assessment via TICSm: IORW (N = 9,385) eFigure. Sample selection flowchart eReference [file jamanetwopen-e2519622-s001.pdf]

Supplemental Online Content

Zhou Z, Hong X. Caring for grandchildren and dementia among older adults in China. *JAMA Netw Open*. 2025;8(7):e2519622.
doi:10.1001/jamanetworkopen.2025.19622

eMethods. Marginal structural models (MSMs) and inverse odds ratio weighting
(IORW) analysis
eTable 1. Summary of Time-varying Confounders according to Grandchild Care in the Weighted Population
eTable 2. Association of Grandchild Care with Mobile Phone Ownership, Broadband Internet Access, and Low Loneliness in Older Chinese Adults: MSMs (N = 10,058) **eTable 3.** Mediation Analyses of the Association between Grandchild Care and Dementia Risk via MSMs with 5th/95th Truncated Weights (N = 10,058)
eTable 4. Mediation Analyses of the Association between Grandchild Care and Dementia Risk via MSMs with 10th/90th Truncated Weights (N = 10,058)
eTable 5. E values of the Association between Grandchild Care and Dementia Risk in Older Chinese Adults (N = 10,058)
eTable 6. Mediating Roles of Mobile Phone Ownership, Broadband Internet Access, and Low Loneliness on Dementia Risk via Intensive Grandchild Care Defined as 30 or More: IORW (N = 10,058)
eTable 7. Mediating Roles of Mobile Phone Ownership, Broadband Internet Access, and Low Loneliness on Dementia Risk via Intensive Grandchild Care Defined by Upper Tertile Cutoff: IORW (N = 10,058)
eTable 8. Mediating Roles of Mobile Phone Ownership, Broadband Internet Access, and Low Loneliness on Dementia Risk via Intensive Grandchild Care Defined by Upper Quantile Cutoff: IORW (N = 10,058)
eTable 9. Mediating Roles of Mobile Phone Ownership, Internet Usage, and Low Loneliness on the Association between Grandchild Care and Dementia Risk: IORW (N = 10,058)
eTable 10. Mediating Roles of Mobile Phone Ownership, Broadband Internet Access, and Low Loneliness on Dementia Risk among Participants with Dementia Assessment via TICSm: IORW (N = 9,385)
eFigure. Sample selection flowchart
eReference

This supplemental material has been provided by the authors to give readers additional information about their work.

© 2025 Zhou Z et al. *JAMA Network Open*.

eMethods. Marginal structural models (MSMs) and inverse odds ratio weighting (IORW) analysis

Marginal structural models (MSMs) analysis

Initially, inverse probability weighting (IPW) was used to create a pseudo-population where time-varying confounders were balanced across different levels of grandchild caregiving. Separate IPWs were calculated for exposure (A), censoring (C), and mediators (M) as follows:

$$IPW_i^C = \frac{P(C=0|A=a,F=f_i)}{P(C=0|A=a_i,F=f_i,L=l_i)} \quad (1)$$

$$IPW_i^A = \frac{P(A=a_i|F=f_i)}{P(A=a_i|F=f_i,L=l_i)} \quad (2)$$

$$IPW_i^M = \frac{P(M=m_i|A=a_i,F=f_i)}{P(M=m_i|A=a_i,F=f_i,L=l_i)} \quad (3)$$

Here, A denotes grandchild caregiving, C=0 represents uncensored subjects, M denotes each mediator (mobile phone ownership, broadband internet access, or loneliness), F signifies time-invariant confounders, and L denotes time-varying confounders. Weights were stabilized by truncating at the 1st and 99th percentiles to mitigate the influence of extreme weights. The censoring weight IPW_i^C accounts for potential selection bias arising from censoring due to death or loss to follow-up.

In the second stage, we conducted three weighted logistic regression models to estimate:

(1) The association between grandchild caregiving and dementia risk (Equation 4), weighted by $IPW_i^A \times IPW_i^C$

(2) the association of grandchild caregiving with each mediator (Equation 5), weighted by IPW_i^A

(3) the association of mediators with dementia risk (Equation 6), weighted by $IPW_i^A \times IPW_i^M \times IPW_i^C$

$$logit\ p(Y=1|A=a,F=f) = \alpha_0 + \alpha_1 A + \alpha_2 f \quad (4)$$

$$logit\ p(M=1|A=a,F=f) = \beta_0 + \beta_1 A + \beta_2 f \quad (5)$$

$$logit\ p(Y=1|A=a,M=m,F=f) = \gamma_0 + \gamma_1 A + \gamma_2 M + \gamma_3 f \quad (6)$$

This method is particularly well suited for longitudinal studies with confounders, allowing for the estimation of associations while minimizing bias.

Inverse odds ratio weighting (IORW) analysis

This approach allowed us to decompose the total effect (TE) of grandchild caregiving on dementia into natural direct effects (NDE), reflecting the association independent of the mediators, and natural indirect effects (NIE), representing the pathways operating through mobile phone ownership, broadband internet access, and low loneliness. IORW weights were derived by calculating the inverse of the odds ratios obtained from logistic regression models assessing the association between grandchild caregiving and each mediator, adjusting for relevant confounders. These weights were then applied in separate logistic regression models to estimate the NDE of grandchild caregiving on dementia risk, effectively isolating the pathway independent of the mediators. The NIE for each mediator was subsequently calculated by subtracting the NDE from the TE. The proportion mediated was derived by dividing the NIE by the TE, providing a measure of the relative contribution of each indirect pathway. All analyses were conducted using bootstrapping with 1,000 replications to generate robust confidence intervals for the estimated coefficients.

© 2025 Zhou Z et al. JAMA Network Open.

eTable 1. Summary of Time-varying Confounders according to Grandchild Care in the Weighted Population

Confounders	Grandchild Care, No. (%)					*P* Value[a]
	Total	Noncaregivers	Nonintensive caregivers	Intensive caregivers	Having no grandchild	
Rural residence	31091.6(77.0)	7754.9(77.1)	7680.5(76.7)	7669.3(76.7)	7986.9(77.5)	0.75
Married	36081.5(89.4)	8962.5(89.1)	8951.6(89.4)	8981.8(89.9)	9185.6(89.1)	0.88
Education						
Non-literate	9313.9(23.1)	2253.5(22.4)	2260.5(22.6)	2154.9(21.6)	2645.1(25.7)	
Elementary school	16950.3(42.0)	4302.3(42.8)	4204.2(42.0)	4316.4(43.2)	4127.4(40.0)	0.19
Middle school or above	14109.9(34.9)	3502.1(34.8)	3547.5(35.4)	3525.1(35.3)	3535.2(34.3)	
Current working	28136.6(69.7)	7085.9(70.5)	6997.7(69.9)	7068.4(70.7)	6984.6(67.8)	0.11
Log-transformed income[b]	8.0(2.4)	8.0(2.3)	8.0(2.3)	8.0(2.3)	8.0(2.5)	0.38[c]
Living arrangement						
Living with spouse only	11635.5(28.8)	3317.8(33.0)	2454.6(24.5)	2212.7(22.1)	3650.4(35.4)	
Skipped-generation	4670.6(11.6)	354.3(3.5)	1822.3(18.2)	2145.9(21.5)	348(3.4)	0.34
Living alone	1856.5(4.6)	711.3(7.1)	266.4(2.7)	227.9(2.3)	651(6.3)	
Other	22211.5(55.0)	5674.4(56.4)	5468.9(54.6)	5409.8(54.1)	5658.3(54.9)	
Smoking status						
Nonsmoker	22188(55.0)	5475(54.4)	5544.9(55.4)	5477.5(54.8)	5690.5(55.2)	
Former smoker	2859.9(7.1)	796.9(7.9)	659.1(6.6)	670.8(6.7)	733.1(7.1)	0.68
Current smoker	15326.2(38.0)	3785.9(37.6)	3808.2(38.0)	3848(38.5)	3884.1(37.7)	
Drinking status						
Nondrinker	21584.9(53.5)	5358.1(53.3)	5352.7(53.5)	5288.9(52.9)	5585.2(54.2)	
Former drinker	4281.9(10.6)	1062.3(10.6)	993.8(9.9)	1127.9(11.3)	1097.9(10.7)	0.67
Current drinker	14507.3(35.9)	3637.4(36.2)	3665.7(36.6)	3579.5(35.8)	3624.6(35.2)	
Sleep duration per night, h						
-6	13748.2(34.1)	3503.9(34.8)	3383.5(33.8)	3349.5(33.5)	3511.3(34.1)	
6-9	24230.1(60.0)	5890.1(58.6)	6074.2(60.7)	6125.5(61.3)	6140.4(59.6)	0.76
9-	2395.7(5.9)	663.8(6.6)	554.5(5.5)	521.4(5.2)	656(6.4)	
Medical insurance	39146.1(97.0)	9739.8(96.8)	9713.4(97.0)	9704.6(97.1)	9988.3(96.9)	0.88
Chronic conditions	29508.3(73.1)	7328.6(72.9)	7322.3(73.1)	7307.3(73.1)	7550.1(73.2)	0.79
ADL Disabled	5002.4(12.4)	1232.8(12.3)	1235(12.3)	1206.2(12.1)	1328.5(12.9)	0.63

Abbreviation: ADL, activities of daily living.

[a] *P* values were calculated from Wald Chi-squared test of the weighted logistics model or weighted multinomial logistics models with each of time-varying confounders as dependent variable and exposure as single independent variable.

[b] Mean (SD)

[c] *P* values were calculated from *F* test of the weighted linear regression model with income as dependent variable and exposure as single independent variable.

© 2025 Zhou Z et al. JAMA Network Open.

eTable 2. Association of Grandchild Care with Mobile Phone Ownership, Broadband Internet Access, and Low Loneliness in Older Chinese Adults: MSMs (N =10,058)

**Variables**	**Odds Ratio (95% CI)** [a]		
	**Mobile Phone Ownership**	**Broadband Internet Access**	**Low Loneliness**
Non-intensive Grandchild care	1.33 (1.06,1.67)	1.34 (1.10,1.62)	1.26 (1.04,1.52)
Intensive Grandchild care	1.55 (1.24,1.93)	1.27 (1.07,1.52)	1.17 (0.99,1.38)
Having No Grandchild	1.01 (0.81,1.26)	1.45 (1.25,1.68)	1.00 (0.83,1.20)
Age	0.34 (0.31,0.38)	0.61 (0.56,0.67)	0.82 (0.75,0.90)
Men	1.31 (1.12,1.54)	1.01 (0.89,1.15)	1.57 (1.38,1.79)
Ethnic Han	1.22 (0.92,1.63)	1.95 (1.48,2.57)	1.26 (0.99,1.60)
Constant	16.36 (11.79,22.69)	0.25 (0.18,0.33)	3.94 (3.00,5.16)

Abbreviation: MSMs, marginal structural models.

[a] Stabilized weights accounting for residency, marital status, education, current working status, income, living arrangements, smoking status, drinking status, sleep duration, medical insurance, chronic conditions, and ADL disabilities.

© 2025 Zhou Z et al. *JAMA Network Open.*

eTable 3. Mediation Analyses of the Association between Grandchild Care and Dementia Risk via MSMs with $5^{th}/95^{th}$ Truncated Weights: MSMs (*N*=10,058)

**Variables**	**Odds Ratio (95% CI)** [a]				
	**Model 1**	**Model 2**	**Model 3**	**Model 4**	**Model 5**
Non-intensive Grandchild care	0.68 (0.54,0.87)	0.72 (0.57,0.91)	0.72 (0.57,0.91)	0.70 (0.55,0.89)	0.75 (0.59,0.95)
Intensive Grandchild care	0.82 (0.66,1.01)	0.84 (0.68,1.04)	0.85 (0.68,1.05)	0.83 (0.67,1.03)	0.87 (0.70,1.08)
Having No Grandchild	1.04 (0.82,1.31)	1.07 (0.84,1.36)	1.11 (0.88,1.41)	1.06 (0.83,1.34)	1.14 (0.89,1.45)
Mobile Phone Ownership	NA	0.55 (0.44,0.69)	NA	NA	0.60 (0.47,0.75)
Broadband Internet Access	NA	NA	0.43 (0.34,0.56)	NA	0.42 (0.32,0.54)
Low Loneliness	NA	NA	NA	0.58 (0.47,0.71)	0.64 (0.52,0.78)
Age	1.92 (1.71,2.15)	1.79 (1.60,2.02)	1.82 (1.62,2.05)	1.91 (1.70,2.15)	1.68 (1.50,1.90)
Men	0.48 (0.40,0.57)	0.48 (0.41,0.58)	0.49 (0.41,0.58)	0.50 (0.42,0.60)	0.50 (0.41,0.59)
Ethnic Han	0.62 (0.47,0.81)	0.62 (0.47,0.81)	0.66 (0.50,0.86)	0.62 (0.48,0.81)	0.68 (0.52,0.89)
Constant	0.12 (0.09,0.17)	0.22 (0.15,0.31)	0.14 (0.10,0.18)	0.18 (0.13,0.25)	0.33 (0.22,0.48)

Abbreviation: MSMs, marginal structural models; NA, not applicable.

[a] Stabilized weights accounting for residency, marital status, education, current working status, income, living arrangements, smoking status, drinking status, sleep duration, medical insurance, chronic conditions, and ADL disabilities.

© 2025 Zhou Z et al. *JAMA Network Open*.

eTable 4. Mediation Analyses of the Association between Grandchild Care and Dementia Risk via MSMs with 10th/90th Truncated Weights: MSMs (N=10,058)

**Variables**	**Odds Ratio (95% CI)** [a]				
	**Model 1**	**Model 2**	**Model 3**	**Model 4**	**Model 5**
Non-intensive Grandchild care	0.67 (0.53,0.85)	0.70 (0.55,0.89)	0.71 (0.56,0.90)	0.69 (0.54,0.87)	0.73 (0.58,0.93)
Intensive Grandchild care	0.80 (0.65,0.99)	0.83 (0.67,1.03)	0.84 (0.67,1.04)	0.82 (0.66,1.01)	0.86 (0.70,1.06)
Having No Grandchild	0.96 (0.76,1.21)	0.98 (0.77,1.24)	1.04 (0.82,1.33)	0.98 (0.77,1.24)	1.06 (0.83,1.34)
Mobile Phone Ownership	NA	0.51 (0.41,0.64)	NA	NA	0.56 (0.45,0.70)
Broadband Internet Access	NA	NA	0.43 (0.33,0.55)	NA	0.41 (0.32,0.53)
Low Loneliness	NA	NA	NA	0.58 (0.47,0.70)	0.63 (0.52,0.77)
Age	1.92 (1.71,2.15)	1.77 (1.57,1.98)	1.83 (1.63,2.05)	1.91 (1.71,2.15)	1.66 (1.48,1.87)
Men	0.47 (0.40,0.56)	0.48 (0.40,0.57)	0.48 (0.40,0.57)	0.49 (0.41,0.59)	0.49 (0.41,0.58)
Ethnic Han	0.62 (0.48,0.81)	0.63 (0.48,0.82)	0.67 (0.51,0.87)	0.63 (0.48,0.82)	0.69 (0.53,0.89)
Constant	0.12 (0.09,0.17)	0.24 (0.16,0.34)	0.14 (0.10,0.19)	0.18 (0.13,0.25)	0.36 (0.24,0.52)

Abbreviation: MSMs, marginal structural models; NA, not applicable.

[a] Stabilized weights accounting for residency, marital status, education, current working status, income, living arrangements, smoking status, drinking status, sleep duration, medical insurance, chronic conditions, and ADL disabilities.

© 2025 Zhou Z et al. *JAMA Network Open*.

eTable 5. *E* values of the Association between Grandchild Care and Dementia Risk in Older Chinese Adults (*N* = 10,058)

	E-Value (CI Limit)[a]				
**Variables**	**Model 1**[b]	**Model 2**[c]	**Model 3**[d]	**Model 4**[e]	**Model 5**[f]
Non-intensive Grandchild care	2.26 (1.53)	2.04 (1.32)	2.08 (1.39)	2.17 (1.46)	1.96 (1.21)
Mobile Phone Ownership	NA	2.40 (1.67)	NA	NA	2.21 (1.46)
Broadband Internet Access	NA	NA	3.87(2.90)	NA	4.08 (2.90)
Low Loneliness	NA	NA	NA	2.84 (2.17)	2.50 (1.88)

Abbreviation: NA, not applicable.

[a] See VanderWeele and Ding[1] for the formula for calculating E-values.

[b] Model 1 adjusted for age, men, and ethnic han.

[c] Model 2 further adjusted for mobile phone ownership based on model 1.

[d] Model 3 further adjusted for broadband internet access based on model 1.

[e] Model 4 further adjusted for low loneliness based on model 1.

[f] Model 5 further adjusted for mobile phone ownership, broadband internet access, low loneliness based on model 1.

© 2025 Zhou Z et al. *JAMA Network Open.*

eTable 6. Mediating Roles of Mobile Phone Ownership, Broadband Internet Access, and Low Loneliness on Dementia Risk via Intensive Grandchild Care Defined as 30 or More: IORW (*N* = 10,058)

Exposure	Mediators	Odds Ratio (95% CI) [a]			Mediation proportion % (95% CI) [b]
		TE	NDE	NIE	
Non-intensive Grandchild care	Mobile Phone Ownership (M_1)	0.70 (0.65, 0.74)	0.74 (0.67, 0.80)	0.95 (0.89, 0.99)	15.36 (1.99, 31.19)
	Broadband Internet Access (M_2)	0.70 (0.65, 0.74)	0.74 (0.68, 0.79)	0.94 (0.91, 0.98)	15.79 (5.35, 27.18)
	Low loneliness (M_3)	0.70 (0.65, 0.74)	0.74 (0.68, 0.79)	0.95 (0.91, 0.98)	15.54 (4.67, 26.58)
	M_1 + M_2 + M_3	0.70 (0.65, 0.74)	0.82 (0.76, 0.87)	0.85 (0.82, 0.89)	43.50 (31.92, 55.95)
Intensive Grandchild care	Mobile Phone Ownership (M_1)	0.81 (0.66,1.01)	0.86 (0.67,1.11)	0.94 (0.91, 0.99)	28.22 (3.81, 50.41)
	Broadband Internet Access (M_2)	0.81 (0.66,1.01)	0.82 (0.66,1.03)	0.98 (0.96, 1.02)	7.97 (-6.81, 22.84)
	Low Loneliness (M_3)	0.81 (0.66,1.01)	0.82 (0.65,1.03)	0.99 (0.96, 1.02)	5.57 (-9.64, 21.53)
	M_1 + M_2 + M_3	0.81 (0.66,1.01)	0.82 (0.66,1.01)	0.99 (0.96, 1.03)	3.96 (-14.43, 19.58)
Having No Grandchild	Mobile Phone Ownership (M_1)	1.11 (0.87,1.40)	1.10 (0.83,1.46)	1.01 (0.97, 1.05)	6.31 (-45.17, 86.09)
	Broadband Internet Access (M_2)	1.11 (0.87,1.40)	1.14 (0.88,1.49)	0.97 (0.94, 1.00)	-30.18 (-113.96, 0.32)
	Low loneliness (M_3)	1.11 (0.87,1.40)	1.13 (0.86,1.47)	0.98 (0.95, 1.01)	-15.32 (-75.97, 20.53)
	M_1 + M_2 + M_3	1.11 (0.87,1.40)	1.08 (0.86,1.36)	1.03 (0.99, 1.07)	25.19 (-26.42, 76.84),

Abbreviation: IORW, inverse odds ratio weighted models; TE, total effect; NDE, natural direct effects; NIE, natural indirect effects.

[a] Stabilized weights accounting for residency, marital status, education, current working status, income, living arrangements, smoking status, drinking status, sleep duration, medical insurance, chronic conditions, and ADL disabilities.

[b] Calculated as [NIE / (NDE + NIE)] ×100%.

© 2025 Zhou Z et al. *JAMA Network Open.*

eTable 7. Mediating Roles of Mobile Phone Ownership, Broadband Internet Access, and Low Loneliness on Dementia Risk via Intensive Grandchild Care Defined by Upper Tertile Cutoff: IORW (N = 10,058)

Exposure	Mediators	Odds Ratio (95% CI) [a]			Mediation proportion % (95% CI) [b]
		TE	NDE	NIE	
Non-intensive Grandchild care	Mobile Phone Ownership (M_1)	0.65 (0.52,0.81)	0.69 (0.53,0.91)	0.95 (0.89, 1.00)	13.15 (0.19, 27.62)
	Broadband Internet Access (M_2)	0.65 (0.52,0.81)	0.69 (0.54,0.88)	0.94 (0.91, 0.98)	14.15 (5.16, 23.23)
	Low Loneliness (M_3)	0.65 (0.52,0.81)	0.69 (0.55,0.88)	0.94 (0.91, 0.98)	14.49 (5.35, 23.87)
	$M_1 + M_2 + M_3$	0.65 (0.52,0.81)	0.73 (0.58,0.91)	0.90 (0.86, 0.94)	25.34 (15.65, 35.77)
Intensive Grandchild care	Mobile Phone Ownership (M_1)	0.94 (0.74,1.18)	0.96 (0.73,1.27)	0.97 (0.92, 1.03)	37.82 (-184.12, 485.94)
	Broadband Internet Access (M_2)	0.94 (0.74,1.18)	0.92 (0.72,1.18)	1.02 (0.97, 1.06)	-26.07 (-374.28, 113.12)
	Low Loneliness (M_3)	0.94 (0.74,1.18)	0.91 (0.71,1.17)	1.03 (0.98, 1.08)	-38.75 (-454.86, 185.63)
	$M_1 + M_2 + M_3$	0.9 4(0.74,1.18)	0.93 (0.73,1.17)	1.01 (0.96, 1.06)	-18.23 (-330.55, 176.25)
Having No Grandchild	Mobile Phone Ownership (M_1)	1.11 (0.87,1.40)	1.10 (0.83,1.46)	1.01 (0.95, 1.06)	6.44 (-69.81, 115.24)
	Broadband Internet Access (M_2)	1.11 (0.87,1.40)	1.14 (0.87,1.49)	0.97 (0.93, 1.01)	-28.26 (-135.05, 11.94)
	Low Loneliness (M_3)	1.11 (0.87,1.40)	1.12 (0.86,1.47)	0.99 (0.94, 1.03)	-13.40 (-99.29, 39.08)
	$M_1 + M_2 + M_3$	1.11 (0.87,1.40)	1.08 (0.86,1.36)	1.03 (0.98, 1.07)	25.20 (-49.47, 107.62)

Abbreviation: IORW, inverse odds ratio weighted models; TE, total effect; NDE, natural direct effects; NIE, natural indirect effects.

[a] Stabilized weights accounting for residency, marital status, education, current working status, income, living arrangements, smoking status, drinking status, sleep duration, medical insurance, chronic conditions, and ADL disabilities.

[b] Calculated as [NIE / (NDE + NIE)] ×100%.

© 2025 Zhou Z et al. *JAMA Network Open*.

eTable 8. Mediating Roles of Mobile Phone Ownership, Broadband Internet Access, and Low Loneliness on Dementia Risk via Intensive Grandchild Care Defined by Upper Quantile Cutoff: IORW (*N* = 10,058)

**Exposure**	**Mediators**	**Odds Ratio (95% CI)** [a]			**Mediation proportion % (95% CI)** [b]
		**TE**	**NDE**	**NIE**	
Non-intensive Grandchild care	Mobile Phone Ownership (M_1)	0.70 (0.57,0.86)	0.72 (0.56,0.94)	0.97 (0.92, 1.02)	8.71 (-5.64, 25.20)
	Broadband Internet Access (M_2)	0.70 (0.57,0.86)	0.73 (0.58,0.92)	0.96 (0.93, 1.00)	11.41 (0.70, 21.69)
	Low Loneliness (M_3)	0.70 (0.57,0.86)	0.73 (0.58,0.92)	0.96 (0.93, 1.00)	11.03 (0.42, 21.57)
	$M_1 + M_2 + M_3$	0.70 (0.57,0.86)	0.77 (0.62,0.95)	0.91 (0.88, 0.96)	25.23 (13.53, 36.96)
Intensive Grandchild care	Mobile Phone Ownership (M_1)	0.88 (0.68,1.14)	0.96 (0.71,1.32)	0.92 (0.87, 1.01)	70.82 (-5.80, 215.11)
	Broadband Internet Access (M_2)	0.88 (0.68,1.14)	0.90 (0.68,1.19)	0.98 (0.93, 1.03)	13.46 (-31.91, 70.34)
	Low Loneliness (M_3)	0.88 (0.68,1.14)	0.89 (0.68,1.18)	0.99 (0.94, 1.04)	7.35 (-43.23, 58.32)
	$M_1 + M_2 + M_3$	0.88 (0.68,1.14)	0.91 (0.71,1.17)	0.97 (0.92, 1.03)	21.98 (-30.14, 79.40)
Having No Grandchild	Mobile Phone Ownership (M_1)	1.11 (0.87,1.41)	1.10 (0.82,1.46)	1.01 (0.96, 1.06)	9.16 (-60.66, 103.30)
	Broadband Internet Access (M_2)	1.11 (0.87,1.41)	1.14 (0.87,1.49)	0.97 (0.93, 1.01)	-26.56 (-132.18, 13.76)
	Low Loneliness (M_3)	1.11 (0.87,1.41)	1.12 (0.86,1.47)	0.99 (0.94, 1.03)	-12.06 (-91.97, 40.80)
	$M_1 + M_2 + M_3$	1.11 (0.87,1.41)	1.08 (0.85,1.36)	1.03 (0.98, 1.08)	27.79 (-36.40, 103.61)

Abbreviation: IORW, inverse odds ratio weighted models; TE, total effect; NDE, natural direct effects; NIE, natural indirect effects.

[a] Stabilized weights accounting for residency, marital status, education, current working status, income, living arrangements, smoking status, drinking status, sleep duration, medical insurance, chronic conditions, and ADL disabilities.

[b] Calculated as [NIE / (NDE + NIE)] ×100%.

© 2025 Zhou Z et al. *JAMA Network Open*.

eTable 9. Mediating Roles of Mobile Phone Ownership, Internet Usage, and Low Loneliness on the Association between Grandchild Care and Dementia Risk: IORW (*N* = 10,058)

Exposure	Mediators	Odds Ratio (95% CI) [a]			Mediation proportion % (95% CI) [b]
		TE	NDE	NIE	
Non-intensive Grandchild care	Mobile Phone Ownership (M_1)	0.69 (0.54,0.87)	0.74 (0.55,0.99)	0.93 (0.88, 0.99)	18.22 (2.42, 35.88)
	Broadband Internet Access (M_2)	0.69 (0.54,0.87)	0.73 (0.56,0.95)	0.95 (0.91, 0.99)	15.32 (4.02, 26.83)
	Low Loneliness (M_3)	0.69 (0.54,0.87)	0.73 (0.56,0.96)	0.94 (0.90, 0.98)	16.88 (4.92, 28.74)
	M_1 + M_2 + M_3	0.69 (0.54,0.87)	0.79 (0.62,1.00)	0.88 (0.84, 0.92)	35.95 (23.72, 49.89)
Intensive Grandchild care	Mobile Phone Ownership (M_1)	0.82 (0.66,1.01)	0.84 (0.65,1.09)	0.97 (0.93, 1.03)	14.65 (-13.84, 38.81)
	Broadband Internet Access (M_2)	0.82 (0.66,1.01)	0.81 (0.64,1.01)	1.02 (0.98, 1.06)	-8.90 (-31.46, 9.21)
	Low Loneliness (M_3)	0.82 (0.66,1.01)	0.81 (0.65,1.03)	1.01 (0.97, 1.05)	-3.39 (-25.53, 14.83)
	M_1 + M_2 + M_3	0.82 (0.66,1.01)	0.82 (0.66,1.01)	1.00 (0.96, 1.05)	-0.37 (-28.34, 19.59)
Having No Grandchild	Mobile Phone Ownership (M_1)	1.10 (0.87,1.40)	1.10 (0.83,1.46)	1.01 (0.95, 1.06)	6.86 (-68.20, 132.57)
	Broadband Internet Access (M_2)	1.10 (0.87,1.40)	1.14 (0.87,1.48)	0.97 (0.93, 1.01)	-28.41 (-149.86, 20.08)
	Low Loneliness (M_3)	1.10 (0.87,1.40)	1.12 (0.86,1.47)	0.99 (0.94, 1.03)	-14.82 (-107.88, 45.27)
	M_1 + M_2 + M_3	1.10 (0.87,1.40)	1.07 (0.85,1.35)	1.03 (0.98, 1.08)	31.62 (-46.30, 132.28)

Abbreviation: IORW, inverse odds ratio weighted models; TE, total effect; NDE, natural direct effects; NIE, natural indirect effects.

[a] Stabilized weights accounting for residency, marital status, education, current working status, income, living arrangements, smoking status, drinking status, sleep duration, medical insurance, chronic conditions, and ADL disabilities.

[b] Calculated as [NIE / (NDE + NIE)] ×100%.

© 2025 Zhou Z et al. *JAMA Network Open*.

eTable 10. Mediating Roles of Mobile Phone Ownership, Broadband Internet Access, and Low Loneliness on Dementia Risk among Participants with Dementia Assessment via TICS-m: IORW (*N* = 9,385)

**Exposure**	**Mediators**	**Odds Ratio (95% CI)** [a]			**Mediation proportion % (95% CI)** [b]
		**TE**	**NDE**	**NIE**	
Non-intensive Grandchild care	Mobile Phone Ownership (M_1)	0.72 (0.66, 0.79)	0.78 (0.70, 0.87)	0.93 (0.87, 0.99)	23.53 (2.79, 47.59)
	Broadband Internet Access (M_2)	0.72 (0.66, 0.79)	0.78 (0.70, 0.85)	0.93 (0.89, 0.98)	21.53 (6.19, 39.51)
	Low Loneliness (M_3)	0.72 (0.66, 0.79)	0.78 (0.70, 0.85)	0.94 (0.89, 0.98)	20.93 (5.59, 38.62)
	$M_1 + M_2 + M_3$	0.72 (0.66, 0.79)	0.83 (0.76, 0.91)	0.87 (0.83, 0.92)	42.44 (26.70, 61.61)
Intensive Grandchild care	Mobile Phone Ownership (M_1)	0.82 (0.66,1.03)	0.84 (0.64,1.11)	0.97 (0.93, 1.03)	13.65 (-18.33, 44.55)
	Broadband Internet Access (M_2)	0.82 (0.66,1.03)	0.83 (0.65,1.06)	0.99 (0.95, 1.04)	4.11 (-20.40, 27.29)
	Low Loneliness (M_3)	0.82 (0.66,1.03)	0.82 (0.64,1.05)	1.00 (0.96, 1.05)	-0.49 (-26.84, 21.72)
	$M_1 + M_2 + M_3$	0.82 (0.66,1.03)	0.82 (0.65,1.03)	1.01 (0.96, 1.06)	-3.83 (-34.64, 19.62)
Having No Grandchild	Mobile Phone Ownership (M_1)	1.09 (0.85,1.41)	1.09 (0.80,1.49)	1.00 (0.94, 1.06)	-1.23 (-157.02, 208.69)
	Broadband Internet Access (M_2)	1.09 (0.85,1.41)	1.12 (0.84,1.51)	0.97 (0.93, 1.02)	-27.36 (-219.81, 96.09)
	Low Loneliness (M_3)	1.09 (0.85,1.41)	1.10 (0.82,1.48)	0.99 (0.94, 1.04)	-11.96 (-144.26, 146.44)
	$M_1 + M_2 + M_3$	1.09 (0.85,1.41)	1.05 (0.82,1.35)	1.04 (0.98, 1.10)	41.25 (-81.50, 235.77)

Abbreviation: TICS-m, Telephone Interview for Cognitive Status and its modifications; IORW, inverse odds ratio weighted models; TE, total effect; NDE, natural direct effects; NIE, natural indirect effects.

[a] Stabilized weights accounting for residency, marital status, education, current working status, income, living arrangements, smoking status, drinking status, sleep duration, medical insurance, chronic conditions, and ADL disabilities.

[b] Calculated as [NIE / (NDE + NIE)] ×100%.

© 2025 Zhou Z et al. *JAMA Network Open.*

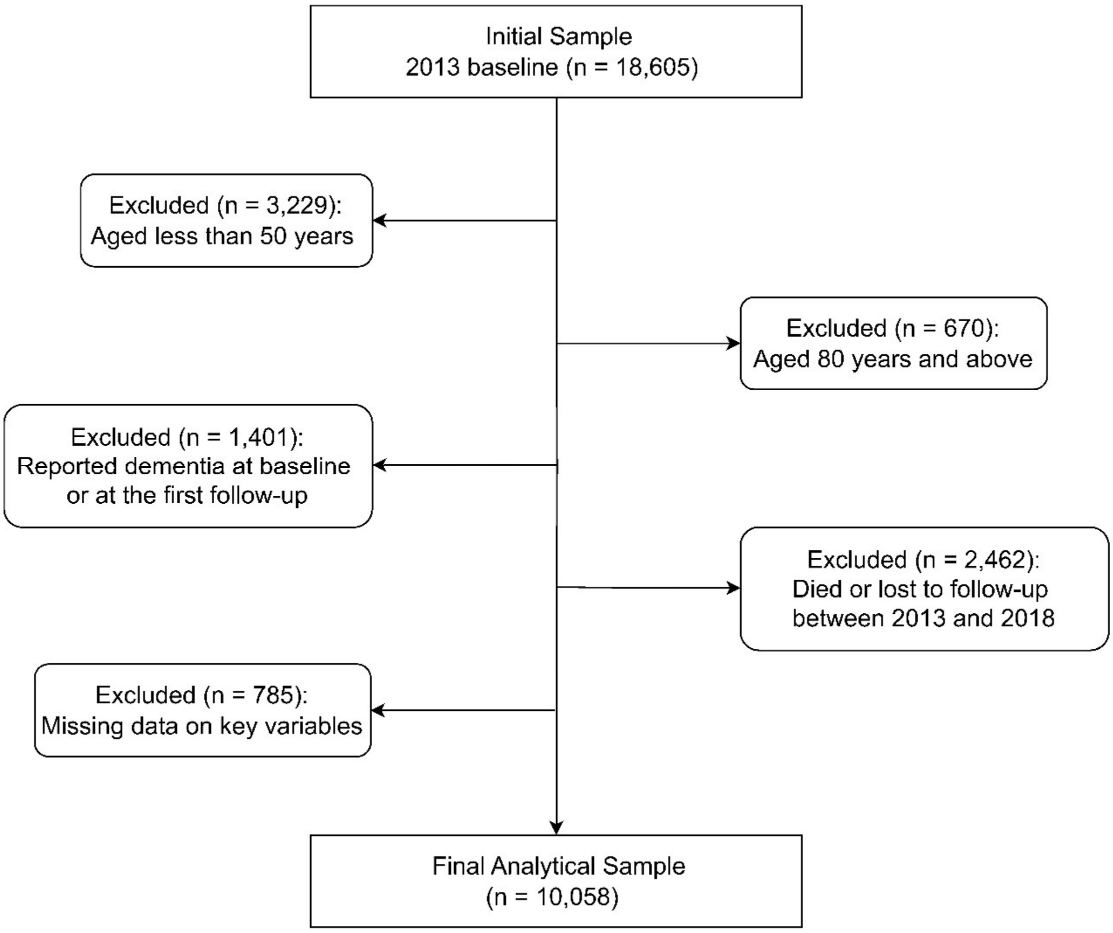

eFigure. Sample selection flowchart

eReference

1. VanderWeele TJ, Ding P. Sensitivity Analysis in Observational Research: Introducing the *E*-Value. *Ann Intern Med* 2017; 167(4): 268-74.

© 2025 Zhou Z et al. *JAMA Network Open*.
