# Supplementary material for: Caring for Grandchildren and Dementia Among Older Adults in China
Source: JAMA Netw Open. 2025 Jul 9;8(7):e2519622. doi: 10.1001/jamanetworkopen.2025.19622 (PMC12242682; doi:10.1001/jamanetworkopen.2025.19622)
Supplement: Supplement 2. — Data Sharing Statement [file jamanetwopen-e2519622-s002.pdf]

Data Sharing Statement

Zhou. Caring for Grandchildren and Dementia Among Older Adults in China. *JAMA Netw Open*. Published July 09, 2025. doi:10.1001/jamanetworkopen.2025.19622

Data
Data available: Yes
Data types: Deidentified participant data
How to access data: https://charls.pku.edu.cn/en/
When available: With publication

Supporting Documents
Document types: None

Additional Information
Who can access the data: anyone can access the data
Types of analyses: any purpose
Mechanisms of data availability: with a signed data access agreement
